# Experimental Nuclear Medicine Meets Tumor Biology

**DOI:** 10.3390/ph15020227

**Published:** 2022-02-14

**Authors:** Theresa Balber, Loan Tran, Katarína Benčurová, Julia Raitanen, Gerda Egger, Markus Mitterhauser

**Affiliations:** 1 Ludwig Boltzmann Institute Applied Diagnostics, 1090 Vienna, Austria; theresa.balber@lbiad.lbg.ac.at (T.B.); loan.tran@lbiad.lbg.ac.at (L.T.); katarina.bencurova@lbiad.lbg.ac.at (K.B.); julia.raitanen@lbiad.lbg.ac.at (J.R.); gerda.egger@meduniwien.ac.at (G.E.); 2 Department for Biomedical Imaging and Image Guided Therapy, Division of Nuclear Medicine, Medical University of Vienna, 1090 Vienna, Austria; 3 Department of Inorganic Chemistry, Faculty of Chemistry, University of Vienna, 1090 Vienna, Austria; 4 Department of Pathology, Medical University of Vienna, 1090 Vienna, Austria

**Keywords:** molecular imaging, molecular pathology, cancer biomarkers, biomarker imaging, tracer development, in vitro models, animal models

## Abstract

Personalized treatment of cancer patients demands specific and validated biomarkers for tumor diagnosis and therapy. The development and validation of such require translational preclinical models that recapitulate human diseases as accurately as possible. Moreover, there is a need for convergence of different (pre)clinical disciplines that openly share their knowledge and methodologies. This review sheds light on the differential perception of biomarkers and gives an overview of currently used models in tracer development and approaches for biomarker discovery.

## 1. Introduction

Cancer is one of the leading public health care issues, which imposes a high clinical burden and requires a vast amount of economic resources [1]. According to the World Health Organization, cancer incidence, prevalence and mortality are expected to increase due to population growth and aging [2]. Therefore, it is of high importance to develop strategies to ensure sustainable healthcare systems, identify patients who may benefit from effective cancer treatment and avoid treatment-related toxicity. One of the ways this can be accomplished is with personalized medicine and cancer biomarkers [3,4]. Personalized diagnosis of diseases in turn strongly depends on the collaboration of different clinical disciplines, which implies the sharing of methods and ideas [5]. This review aims to create a common language between the distinct fields of experimental nuclear medicine and molecular pathology and gives an overview of different approaches for biomarker discovery and currently used models in tracer development.

## 2. Biomarkers

Biomarkers are characteristic biological features that can be objectively measured and indicate a normal biological or pathological process in the body. In literature, a variety of biomarkers derived from molecular, histologic, physiologic or radiographic disease characteristics can be found: blood glucose levels, protein expression, and blood pressure or tumor size, respectively. Even complex organ functions or changes in biological structures are used as medical biomarkers [6].

Furthermore, one distinguishes between diagnostic, prognostic, predictive and therapeutic biomarkers [7,8]. Literally, diagnostic biomarkers are “used to detect or confirm presence of a disease or condition of interest or to identify individuals with a subtype of the disease” [6]. Prognostic biomarkers are patient or tumor characteristics that inform on disease outcome, such as recurrence or progress, independent of treatment. Whereas, predictive biomarkers predict the response of an individual patient to a certain therapy and therefore allow for patient stratification [9]. Patients can thereby be spared from potential toxicity caused by ineffective treatment [8].

The following examples are given to underline these different aspects of biomarkers: Patients with PD-L1 overexpression tend to have a higher response to anti-PD-L1 directed therapy. This is the case especially for non-small cell lung cancer and melanoma. However, the high responses of some patients with low expression levels complicate the use of PD-L1 as an exclusionary predictive biomarker [10]. The value of PD-L1 as a predictive biomarker also varies when addressing different clinical outcomes such as objective response rate, overall survival rate or progression-free survival [11]. Not only the outcome but also the cut-off value needs to be considered carefully [10]. In colorectal cancer, microsatellite instability is a prognostic marker linked to improved survival, likely due to higher infiltration of tumor-infiltrating lymphocytes and M1 macrophages, or lower tumor heterogeneity [12]. Therapeutic biomarkers are biomarkers, used as a target for therapy [8], e.g., vascular growth factors or their receptors are targeted alone or in combination with chemotherapy, to inhibit angiogenesis and affect vascular function [13]. The prostate-specific membrane antigen and its elevated expression is used for diagnosis as well as therapy of prostate cancer but may also potentially play a role as a predictive marker in men with metastatic prostate cancer [14].

### 2.1. Biomarkers in the Context of Nuclear Medicine

In the field of molecular imaging, the term “imaging biomarker” is frequently but often misleadingly used. An imaging biomarker is a measurable biological variable indicative of normal or pathological conditions that is measured using imaging techniques. In accordance with this definition, radiolabeled tracers are not biomarkers themselves but rather tools. Dedicated imaging readouts (e.g., time-activity curves) or (semi-) quantitative parameters such as standardized uptake value or binding potential serve as imaging biomarkers. Due to the non-invasiveness of imaging, imaging biomarkers can be used in sensitive (early) and specific diagnosis of diseases, monitoring of disease progression and therapy response [15].

#### 2.1.1. Molecular Imaging of Biomarkers

Molecular imaging is based on a molecular interaction between an imaging probe (e.g., radiotracer, optical ligand, MR-probe) and a receptor or a characteristic biological feature. In nuclear medicine, this interaction can be detected (and quantified) via the physical decay of the integrated radionuclide by suitable camera technologies, allowing for non-invasive imaging of the whole organism [16,17]. The information obtained by molecular imaging techniques mainly concerns the biological function (or pathological dysfunction) of an organ or tissue [18]. 

Based on the radionuclide, one distinguishes between single-photon emission computed tomography (SPECT; e.g., Tc-99m, In-111) and positron emission tomography (PET; e.g., F-18, Ga-68). Both methods can very sensitively visualize the interactions between targets and ligands and are able to detect picomolar concentrations of radiotracers [19]. Due to the small amount of substance used for diagnostic purposes, undesirable pharmacological effects are very unlikely to occur. Further advantages of PET and SPECT are the unlimited penetration depth of the respective radiation, the possibility of quantification (radioactivity concentration in volumes of interest), and the theoretical unlimited possibilities of application due to the infinity of molecules to be radiolabeled [20]. Challenges of nuclear medicine technologies are the limited spatial resolution, sometimes complex interpretation and analyses of images and higher costs compared to many other diagnostic methods routinely used [21]. Additionally, due to radiation protection issues, repetitive scans might be critical and need to be taken with care.

Multimodal imaging referring to the combination of nuclear medical imaging techniques such as PET and SPECT with magnetic resonance imaging (MRI) or computed tomography (CT), adds the necessary anatomical information for correct organ (or tumor) delineation and image interpretation [22]. CT is based on the differential attenuation of X-rays by tissues of different compositions and densities. It provides anatomical information with high spatial resolution, but rather low soft-tissue contrast. The latter can be improved by the use of contrast agents or can be overcome by using MRI [23]. MRI is based on the measurement of electromagnetic radiation arising from the realignment of excited magnetic moments (usually from protons). The contrast is based on different relaxation times, which are dependent on the chemical composition and surrounding of the respective tissues [24].

Especially in the diagnosis and treatment of cancer, it is essential to evaluate the extent and localization of the primary tumor and potential metastases, tumor heterogeneity or early treatment response. The different imaging modalities are used in clinical practice to guide surgery, biopsy or simply to detect organs affected by the disease. However, the combination of molecular imaging with (image-guided) biopsies, although being invasive, still remains necessary and is extremely valuable (c.f. 2.2).

#### 2.1.2. Radiopharmaceuticals for Biomarker Imaging or Therapy

Radiopharmaceuticals are the heart of nuclear medicine providing the interaction with and quantification of disease-related targets of interest. Mainly, radiopharmaceuticals are radioactively labeled:

- endogenous ligands or derivatives, that map metabolic (dys)functions or
- drugs or (modified) model substances targeting specific disease-related proteins (e.g., enzymes, receptors, transporters) or
- antibodies or antibody constructs directed against specific disease-associated antigens [25].

First generation radiopharmaceuticals are known as radiolabeled compounds, which accumulate in the tissue of interest due to their physical properties and are hence specific but non-targeting in a molecular context. For example, [^99m^Tc]Tc-MIBI, initially designed for cardiac imaging, mimics potassium (K^+^) for transport into cells within well perfused areas with subsequent electrostatic interaction on the mitochondria. However, besides cardiac imaging, applications range from parathyroid or thyroid imaging to breast cancer imaging. Another example is [^99m^Tc]Tc-HEDP, which accumulates in hyperactive bone tissue by interactions of its phosphate groups with hydroxyapatite (“bone-seeking”) and visualizes pathologies with a variety of causes. Accordingly, these tracers visualize a molecular change that is not necessarily causal to the disease [26,27]. In contrast, second generation radiopharmaceuticals bind their target by selective molecular recognition, such as receptor–ligand or antibody–antigen binding (e.g., [^111^In]In-DTPA-Octreotide or [^111^In]In-Zevalin) [28,29].

The type of decay defines the application of the radionuclide for diagnostic or therapeutic purposes. When choosing the radionuclide, apart from the appropriate physical decay mode and energy, compatibility of the physical radionuclide half-life and the biological half-life of the targeting molecule needs to be ensured. For imaging, in general, to reduce the patients’ radiation dose, shorter-lived radionuclides are used, whereas for therapy, longer-lived radionuclides are preferred in order to deliver higher energy doses to the target site [30].

Regarding the nature of the element and the related radiochemistry, non-metallic (“organic”) radionuclides (e.g., C-11, F-18) and radiometals (e.g., Tc-99m, In-111) can be distinguished. While organic radionuclides are covalently attached to the targeting molecule, radiometals need complexation using a suitable chelator (e.g., DOTA, DTPA) [31]. If radiolabeling strategies follow the principle of bioisosteric replacement (e.g., replacing hydroxyl groups by fluorine) or isotopic substitution (replacing carbon-12 by carbon-11), the biological authenticity of the resulting tracer is conserved [25,32]. This radiolabeling strategy is commonly used for small molecules as, for example, radiotracers for imaging of the central nervous system (e.g., [^11^C]PIB) [33]. Due to the short physical half-lives of these organic radionuclides, an on-site cyclotron facility is often required for production whereas many radiometals can be obtained from commercially available generators (e.g., Ge-68/Ga-68 generator) [32,34].

Compared to small molecule tracers, radiometal complexes are rather large in size and are therefore mostly conjugated to larger vectors (e.g., peptides such as [^68^Ga]Ga-DOTA-TATE), as chelator-related alterations of biological properties are more tolerated [35]. In contrast to organic radionuclides, radiometals show a larger variety in physical half-life, radiation type and energy of emitted radiation, which makes them suitable for both, SPECT or PET imaging as well as radioligand therapy (Auger electrons, α, β-) [36,37]. In addition, they contribute significantly to the realization of theranostics in nuclear medicine. This approach combines diagnostic imaging and therapy using the same molecule or at least very similar molecules, labeled with different radionuclides or administered at different doses [30]. The radionuclide pair must be properly selected to achieve similar binding properties and in vivo behavior of both, the diagnostic and the therapeutic compound [38,39].

### 2.2. Biomarkers in the Context of Pathology

Classical pathological assessment of diseases such as cancer has been based on histo-morphological evaluation of tissue biopsies or surgically resected tissues. Immunohistochemistry (IHC) remains the gold standard method in pathological assessment and diagnostics, which might be hampered by the lack of well-characterized, specific antibodies applicable for IHC. Furthermore, sampling bias represents a major limitation in comprehensive tumor analysis, which might not represent the complex heterogeneity of the tumor [40,41]. Intra- and intertumoral heterogeneity are common and may contribute to reduced efficacy and therapy resistance. Targeted isolation and detection of tumor cells using biopsies for diagnosis is therefore complicated and might result in sampling errors or complete failure of tumor isolation. Moreover, IHC only provides a static image. These limitations might be circumvented by molecular imaging and new molecular techniques such as liquid biopsies, which hold great promise for improving cancer care [42]. 

Liquid biopsies allow for the assessment of different biomarkers in body fluids including blood, urine, sputum or tissue aspirates. Tumor-derived molecules such as circulating tumor cells (CTCs), cell free circulating tumor DNA (ctDNA), extracellular vesicles, proteins or metabolites are the main basis for the development of biomarkers for cancer patients from liquid biopsies. Besides enumeration of CTCs, the genomic characterization of those cells and ctDNA is promising, to observe the “systemic” tumor characteristics and might be effective for minimally invasive early diagnosis, prognosis, therapy selection, therapy monitoring and follow up. Importantly, several liquid biopsy assays for the assessment of CTCs or genetic alterations in ctDNA were approved by the FDA and have entered the clinics [42].

Together with molecular imaging, a liquid biopsy will enable minimal-invasive and dynamic testing providing more efficient screening, diagnosis, and therapeutic monitoring strategies.

## 3. Biomarker Discovery and Target Identification

Regarding personalized cancer medicine, biomarkers are essential in helping to dissect the heterogeneity of cancers and guiding clinical decision making in oncology. Cancer is a heterogeneous disease not only in terms of its biological behavior but also regarding patient prognosis and response to treatment. On a molecular level, the heterogeneity is displayed on multiple levels: genomics, epigenomics, transcriptomics and proteomics features. Recent clinically implemented biomarkers are mainly based on transcriptomic or genetic tumor characteristics and many have been developed for companion diagnostic use [43,44].

**Figure 1:**
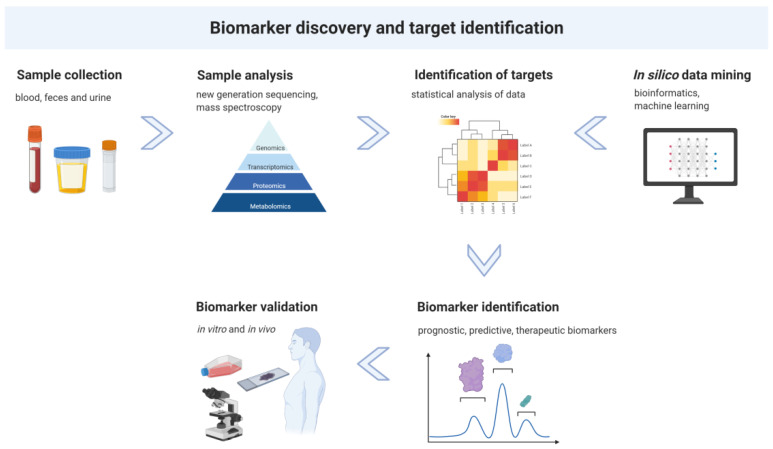
Schematic illustration of the pipeline and interaction of biomarker discovery and target identification. This figure was created with BioRender.com.

In order to define novel biomarkers and imaging targets, it is vital to understand the relationship between measurable biological processes and clinical outcome. Since proteins govern the functions of cells and subcellular compartments, mass spectroscopy has emerged as a powerful tool to study the proteome of healthy volunteers and patients. This approach contributes significantly to the discovery of biomarkers that point to clear indicators of disease (Figure 1) [45,46]. This technology enables system-wide identification and quantification of proteins. Protein signatures encompass not only total levels of expression, but predominance of different isoforms, cleavage products, or post-translational modifications (e.g., phosphorylation or methylation) [45,47]. The characterization of proteomics and metabolomics features provides the basis for the development of reliable imaging as well as molecular biomarkers. 

As an alternative strategy to patient samples, the availability of data from different public databases such as The International Cancer Genome Consortium (ICGC) [48], The Cancer Genome Atlas (TCGA) [49], The Clinical Proteomic Tumor Analysis Consortium (CPTAC) [50], The Cancer Genome Anatomy Project (CGAP) [51], The Cancer Genome Workbench (CGWB) [52] and the Catalogue of Somatic Mutations in Cancer (COSMIC) [53] allows for in silico data mining of existing datasets to discover or validate candidate biomarkers in large cohorts (Figure 1). Furthermore, the use of artificial intelligence approaches including machine learning and deep learning has raised general interest and will help to accelerate data mining and biomarker discovery in the near future both for molecular and imaging-based biomarker discovery [4,54,55,56].

## 4. Experimental Nuclear Medicine

The success story of molecular imaging highly depends on the availability of powerful imaging probes with favorable biodistribution and imaging characteristics. Radiopharmaceuticals must reach the target of interest in a reasonable time period and with a high degree of reproducible metabolic stability. These characteristics of new imaging biomarkers need thorough validation and translation in a systematic way during tracer development to serve as decision-making tools in clinical practice [57].

### 4.1. Binding Studies

Radioligand binding assays are commonly employed during radiopharmaceutical development to determine the affinity and selectivity of a ligand for its respective target, binding kinetics and number of available binding sites. By performing binding assays for several time points, information about association and dissociation rates of the ligand from the receptor is obtained over time and optimal assay conditions for further (saturation) experiments can be acquired [58,59]. 

Semi-automated devices became recently available allowing for the assessment of binding kinetics from a single radioligand concentration in real-time: a detector, mounted on top of an inclined support, measures cell-associated radioactivity on a rotating petri-dish (cell pole, target region) in comparison with a reference region [60]. Thereby, the interaction of a radiotracer with cells of interest (e.g., uptake, binding), but also non-targeted processes such as non-specific binding (to the plastic surface of the dish), can be tracked in real-time [61,62]. 

Receptor saturation and competitive binding experiments are performed at binding equilibrium to determine affinity constants, such as Kd (equilibrium dissociation constant) or Ki (inhibitory constant). The maximal number of available binding sites (Bmax) is determined using receptor-saturating concentrations of radioligand. In addition, blocking or displacement conditions are employed to determine a specific signal by co-incubation with an excess of unlabeled competitor targeting the receptor of interest. These assays can be performed either using whole cells or membrane preparations [59,63]. 

Autoradiography, being amongst the most sensitive imaging techniques, can be performed either in vitro (incubation of tissue sections with radioligand) or ex vivo (tracer injection followed by organ removal and tissue sectioning). The latter involves physiological tracer distribution and in vivo binding, whereby the radioactivity detection is carried out post mortem using dedicated phosphor storage screens or autoradiographic emulsions. Co-injection of (ex vivo) or co-incubation with (in vitro) an excess of unlabeled competitor or inhibitor targeting the receptor of interest reduces the specific signal of the radioligand. Thus, blocking or displacement experiments are used to determine specific binding [64]. 

Deeper insights into pathophysiological pathways of underlying diseases can be obtained by comparison of autoradiograms with immunohistochemical staining of ideally identical or adjacent tissue sections. While autoradiograms reflect radioligand binding to tissues, immunohistochemistry proves the respective target expression. Both however provide only a snapshot, whereas in vivo molecular imaging provides real-time distribution in the living organism, showing how complementing the two different fields are. Since the interaction between tracer and target are comparable to autoradiography or IHC, molecular imaging based on tracers could also be described as in vivo pathology.

### 4.2. In Vitro Models

The above-mentioned assays to evaluate suitable molecular tracers are first and foremost conducted using the already in the 1900s introduced, two-dimensional (2D) cell culture systems [65]. However, although simple, as well as inexpensive, well-established, and reproducible, 2D cell culturing can only limitedly reflect in vivo cell and tissue characteristics. Unnatural cell shape, loss of cell–cell interactions and selection of specific clones, which only partially represent the tumor they originated from, due to prolonged cultivation of cells as monolayers, are only three out of many drawbacks [66].

These drawbacks are expected to be remedied by three-dimensional (3D) cell culture, pioneered by Holtfreter, which has the potential to revolutionize drug testing and disease modeling [67,68]. In contrast to adherent 2D cultures, where cells grow in monolayers, 3D culture is based on spheroid structures emerging from layering cells [69]. The methods used to generate 3D tumor spheroids are either scaffold-based (e.g., hydrogel-based, polymeric hard material-based support or hydrophilic glass fiber) or scaffold-free techniques (e.g., hanging-drop, spheroid microplates with ultra-low attachment coating or magnetic levitation), which are chosen depending on the intended use, as they have different advantages and disadvantages related to the respective method [68].

In general, multicellular tumor spheroids are characterized by a surface layer, consisting of proliferating cells, and an intermediate layer, which is connected to a perinecrotic region. This perinecrotic region contains non-proliferating hypoxic cells, which form a necrotic core—a phenomenon commonly observed when the diameter of the spheroid reaches 500 µm [70,71,72]. 

This structure not only leads to cellular heterogeneity within the spheroid and thus a more realistic mimic of in vivo tumors, but also develops a molecular gradient and penetrating barrier for drugs and metabolites, allowing researchers to conduct drug treatment tests without the use of animals. Nevertheless, 3D culturing methods still face limitations. Amongst them is the difficulty of imaging (e.g., fluorescence imaging with z-stack needed providing only low throughput), and the fact that many assays turn out to be endpoint assays due to the need for generation of single-cell suspensions (e.g., flow cytometry) [73,74]. However, the most important drawback is the lack of vascularization and of the tumor microenvironment, including immune cells, fibroblasts and endothelial cells. 

Some limitations were recently leveraged by the development of organoids, innovative 3D primary cultures that mimic the native organ microstructures and are derived from self-organizing mammalian pluripotent or adult stem cells. Histological and molecular characterization of organoid cultures has shown that organoids recapitulate the structure and function of organs and tissues [71]. Further, organoids derived from human patients have been established and serve as disease models, including cancer. Tumor organoids reproduced the grade and differentiation capacity of their parental tumors to a remarkable degree [75]. Patient-derived tumor organoids (PDOs) hold great promise for personalized cancer medicine, whilst reflecting different levels of inter- and intratumoral heterogeneity seen in cancer patients. Accumulating evidence indicates that PDOs can predict drug and therapy responses in the clinic [76]. Organoids, therefore, represent an advanced model system to study the role of molecular features in cancer and its impact on tumor burden and drug response. For translational-based cancer research, organoid biobanks provide a platform for biomarker testing as well as drug or small molecule screening and have significantly contributed to biomedical as well as basic research. 

PDOs provide a model of human cancer, still representing many characteristics of the original tumor. Organoids can be used to simulate the clinical situation more appropriately, for understanding both, cancer-associated and tracer-related processes. This leads to a more pronounced comprehension of the mechanisms and potential interpretation of the clinical setting. 

### 4.3. In Vivo Models

Despite the great progress in the development of advanced in vitro models, animal models remain indispensable in cancer research. They play a critical role in the transition from fundamental diagnostic and therapeutic discoveries to human clinical trials. Murine models, in particular, contributed significantly to the understanding of the genetic basis of cancer and helped to elucidate the role of specific genes and respective mutations in cancer development and progression [77]. 

Genetically modified rodents and immunocompromised mice engrafted with (human) tumor cell lines allow for studying a variety of different tumor types and imaging of those. However, these models are often criticized for their low translational value and negligible clinical significance, as they reflect human tumor biology only in some aspects [78]. Organoid-based mouse tumor xenograft models allow for more complex tumor characterization, including in vivo drug testing and evaluation of imaging biomarkers [79].

In general, small animal cancer models are more frequently used as (the scarcely used) large animal models. Due to their small size, short lifespan and high fecundity (compared to large animals and humans), these models allow for timely studies, large cohorts, ease of housing and cost-effective maintenance. In this context, the use of non-mammalian vertebrate models in cancer research such as zebrafish has become increasingly popular. Until the onset of the adaptive immune system at around seven days post-fertilization, zebrafish embryos can engraft transplanted cancer cells (zebrafish xenograft models). Moreover, genetic models of cancer in zebrafish have been developed. Aspects of human disease can be recapitulated and followed in vivo at the molecular level because cancer-related genes and signaling pathways are largely evolutionarily conserved between zebrafish and humans [80,81]. However, this model is underexploited in tracer development and nuclear medical imaging.

The chorioallantoic membrane (CAM) assay represents another embryonal, non-mammalian, vertebral model and serves as a potential alternative to rodent models. The highly vascularized chicken CAM allows for tumor cell inoculation, transplantation of tissue specimens, and vascularization of those. The chick embryo is only fully immune-competent shortly before hatching (developmental day 18), reducing the risk of xenograft rejection [82,83,84]. Inoculation and growth of organoids and tissues have successfully been performed contributing to the translational value of this model [85,86,87]. While this model has been used for longer than a century in tumor research and toxicity testing [88], it only gained interest in preclinical imaging in the last decade [89,90]. The distribution of different radiopharmaceuticals in the chicken embryonal organism has been studied and compared to mice, providing the first evidence for the suitability of this model in tracer development [91,92]. Moreover, PET imaging of CAM-grown xenografts has been performed in combination with CT or MRI [92,93,94].

However, developing cancer biomarkers for precision medicine demands highly translational animal models that accurately recapitulate human pathology. Moreover, in the clinics, cancer patients commonly present with multiple morbidities. Ideally, animal models should develop cancer on the background of representative comorbidities such as diabetes [77]. Since swine share a number of anatomic and physiologic characteristics with humans, porcine models became the most important surgical models in biomedical research [95]. In addition, sequencing of the swine genome revealed genetic similarities with humans [96]. A genetically modified porcine model of cancer in which pigs express a mutation in TP53 (which encodes p53) that is orthologous to one commonly found in humans (R175H mutation), represents a large-animal tumor model that replicates the human condition [97]. The Oncopig Cancer Model, a transgenic porcine model for the study of hematologic and solid tumors with mutations in key tumor suppressors and oncogenes, recapitulates transcriptional hallmarks of human tumor biology and exhibits clinically relevant tumor phenotypes [77]. Unfortunately, most research institutions cannot afford the high logistical and housing requirements of large animals. In addition, most preclinical imaging facilities are only equipped with miniature scanners dedicated to small animal imaging, which hampers imaging in large animal models such as the Oncopig.

**Figure 2:**
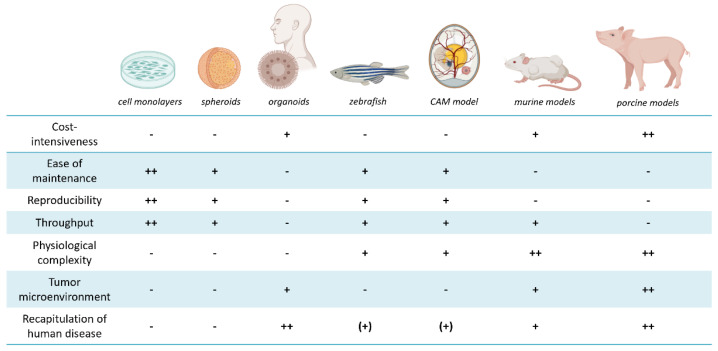
Key aspects of the described in vitro and in vivo models are summarized. The models were rated relative to each other in different categories. Relative scores are presented as best suited ++, suited +, partly suited (+) and not suited −. For example, costs (initial outlay and maintenance costs), as well as care-intensiveness, may play a critical role in the selection of the model. Animal models are generally more laborious and demanding in terms of equipment and care than in vitro models. However, the generation and maintenance of human organoid cultures are relatively expensive as they require specialized reagents and growth factors. The reproducibility of results highly depends on the grade of standardization of an assay or experiment. Cell experiments using 2D cultures can be performed easily in a standardized manner, while experiments based on human material or living organisms usually have higher variability. At the same time, it is hardly feasible to handle large cohorts or sample sizes when experimenting with large animals or very sophisticated models. The translational value of a model not only depends on the physiological complexity or used species but also if the chosen model organism to be engrafted is immunocompromised or immunocompetent, enabling to study the tumor microenvironment. In the context of personalized medicine, patient-derived organoid cultures or PDX animal models recapitulate human disease more accurately than immortalized cell lines or murine xenograft models of those. The figure was partly created with BioRender.com.

The advantages and disadvantages of the described models are manifold and are presented in Figure 2 in a reduced, but clear format.

## 5. Conclusions

A successful establishment and translation of new biomarkers from a preclinical setting towards the clinical application strongly depends on the significance of the target, and the performance and explanatory power of the model in use. We are all aware of the fact that the time for developing and establishing new tracers from bench to bed is far too long. Established models and cell lines, used in the imaging community and validated for our needs for decades, provide limited insights into human disease. Experimental nuclear medicine needs to learn from and to team up with different fields of tumor biology, molecular pathology and oncology.

In a clinical setting, personalized diagnosis of tumor diseases requires the ability and readiness to converge with different fields and disciplines. This need was already recognized by others and Sorace et al. proposed an idealized workflow model for radiology–pathology integration [98]. Not only nuclear medicine but also pathology itself can benefit from molecular information obtained by imaging and a need for molecular imaging-based pathology was identified as well [41]. 

In the publication “Convergence—The Future of Health Report 2016”, the authors state literally that “Convergence comes as a result of the sharing of methods and ideas by chemists, physicists, computer scientists, engineers, mathematicians, and life scientists across multiple fields and industries. It is the integration of insights and approaches from historically distinct scientific and technological disciplines” (5). With this in mind, we wanted to give an overview of different methods and approaches in the fields of experimental nuclear medicine and molecular pathology to create a common understanding and facilitate future collaborations. We expect that functionally and biologically relevant biomarkers will allow for better diagnosis and monitoring of cancer. In addition, the combination of different biomarkers measured by non-invasive approaches such as molecular imaging and liquid biopsies might result in synergies that will eventually foster personalized cancer care.

## Data Availability

Not applicable.
